# Peripheral Hypertrophic Subepithelial Corneal Degeneration

**DOI:** 10.3390/jcm15051681

**Published:** 2026-02-24

**Authors:** Adam Wylęgała, Claudia Azzaro, Patrycja Potrawa, Gabriella De Salvo, Edward Wylęgała, Anna Roszkowska

**Affiliations:** 1 Experimental Ophthalmology Unit, Department of Biophysics, Division of Dentistry, II School of Medicine, Zabrze Medical University of Silesia, 40-752 Katowice, Poland; 2 Ophthalmology Clinic, Department of Biomedical and Dental Sciences and Morphofunctional Imaging, University of Messina, 98122 Messina, Italy; claudia.azzaro@icloud.com (C.A.); aroszkowska@unime.it (A.R.); 3 Ophthalmology Department, Railway Hospital, Zabrze Medical University of Silesia, 40-752 Katowice, Poland; patrycja.potrawa@gmail.com (P.P.); ewylegala@sum.edu.pl (E.W.); 4 Eye Unit, University Hospital Southampton NHS Foundation Trust, Southampton SO16 6YD, UK; gabriella.desalvo@uhs.nhs.uk; 5 Clinical and Experimental Sciences, University of Southampton, Southampton SO16 6YD, UK; 6 Faculty of Medicine, University Andrzej Frycz Modrzewski, 30-705 Krakow, Poland

**Keywords:** peripheral hypertrophic subepithelial corneal degeneration, corneal imaging, optical coherence tomography, corneal topography, corneal astigmatism

## Abstract

**Objectives**: To characterize the clinical features, corneal topography, and imaging findings of peripheral hypertrophic subepithelial corneal degeneration (PHSCD) in a single-center study and to evaluate potential associations with systemic conditions. **Methods**: All patients underwent comprehensive ophthalmic examination, anterior segment photography, high-resolution spectral-domain optical coherence tomography (OCT), and corneal topography/tomography. Patient demographics, ocular history, systemic conditions, and corneal parameters were analyzed. **Results**: Fourteen patients were included in the study (11 females and 3 males). The mean age was 52.6 ± 12.4 years, and the mean best-corrected visual acuity was 0.56 ± 0.23. Five females had Hashimoto’s disease and two had hyperthyroidism. The mean central corneal thickness was 549.4 μm (SD = 71.0 μm), with significant sectoral thickness variations, particularly in the superior-nasal quadrants (SN-IT sector mean difference: 56.4 μm). High-resolution OCT revealed sharply demarcated, hyperreflective fibrosis within the anterior stroma, predominantly in the superior-nasal quadrants, causing corneal flattening with compensatory steepening and astigmatism. Three patients underwent in vivo confocal microscopy, which showed fibrotic acellular tissue adjacent to normal corneal epithelium. **Conclusions**: PHSCD predominantly affects middle-aged females and presents with characteristic peripheral, subepithelial fibrosis, causing significant corneal thickness variations and astigmatism. The observed association with thyroid disorders, particularly Hashimoto’s disease, suggests a potential immunological component in PHSCD pathogenesis that warrants further investigation. Advanced imaging with OCT and confocal microscopy provides valuable diagnostic information to accurately characterize this rare corneal condition.

## 1. Introduction

Peripheral hypertrophic subepithelial corneal degeneration (PHSCD) is a relatively newly identified corneal condition, first described in 2003 [1]. Though its exact etiology remains unclear, it has emerged as a distinct clinical entity, often compared to other well-known corneal disorders, such as Salzmann’s nodular degeneration (SND). PHSCD predominantly occurs bilaterally and is associated with reduced tear secretion and compromised tear film stability [2]. The condition is most seen in women aged between 50 and 60, suggesting a middle-aged demographic as the primary group at risk [3]. The diagnostic criteria for typical PHSCD are increased circumferential and perilimbal subepithelial fibrosis (between the epithelium and Bowman’s layer) associated with focal superficial corneal neovascularization [4]. The disease mostly affects females, is bilateral, and involves the upper corneal quadrants. Induced irregular astigmatism, decreased visual acuity and ocular surface discomfort are the main symptoms and clinical signs of inflammation [1]. PHSCD is considered an idiopathic condition, and its differential diagnosis includes SND, corneal intraepithelial neoplasia, corneal amyloidosis, climatic droplet keratopathy, and keloid. In addition to these pathological conditions, special attention should be given to acquired corneal sub-epithelial hypertrophy (ACSH) reported in patients with paracentral or peripheral superficial corneal opacities after penetrating keratoplasty [5,6]. Given the potential for progressive visual impairment due to the gradual flattening of the central cornea, a notable shift toward hyperopia and irregular astigmatism, early recognition and management of PHSCD is essential [7]. Treatment options, such as superficial keratectomy or phototherapeutic keratectomy (PTK), aim to remove the fibrotic tissue and regularize the corneal profile [3,8]. In severe forms, additional fibrous peeling (FP) and amniotic membrane transplantation may be adopted.

The purpose of this single-center study is to describe a case series of PHSCD to identify the main characteristics, improve the diagnosis, and determine an adequate therapeutic approach.

## 2. Patients and Methods

The study was conducted in accordance with the Declaration of Helsinki and approved by the Board of Ethical Committee of the Medical University of Silesia, Katowice, Poland (approval code: PCN/CBN/0022/KB1/84/21, approval date: 15 June 2021).

In this prospective non-interventional single-center study, 14 subjects (11 females and 3 males) were included. The mean age of the population was 52.6 ± 12.4 years, with a median age of 50.5 years. The minimum age in the sample was 33 years, and the maximum age was 78 years. The patients were enrolled in the Ophthalmology Department of Zabrze Medical University of Silesia in Poland.

The mean best-corrected visual acuity (BCVA) was 0.56 ± 0.23, with a range from 0.20 to 1.00. The sample included patients with a history of various ocular conditions and treatments, including two patients who had undergone cataract surgery, one patient post-photorefractive keratectomy (PRK), one patient with a history of retinitis, and one patient who had received radiotherapy for thyroid cancer. Five females had a medical history of Hashimoto’s disease, while an additional two had hyperthyroidism. Furthermore, seven patients subsequently underwent phototherapeutic keratectomy (PTK). All measurements in this study were conducted before any treatment.

**Figure 1:**
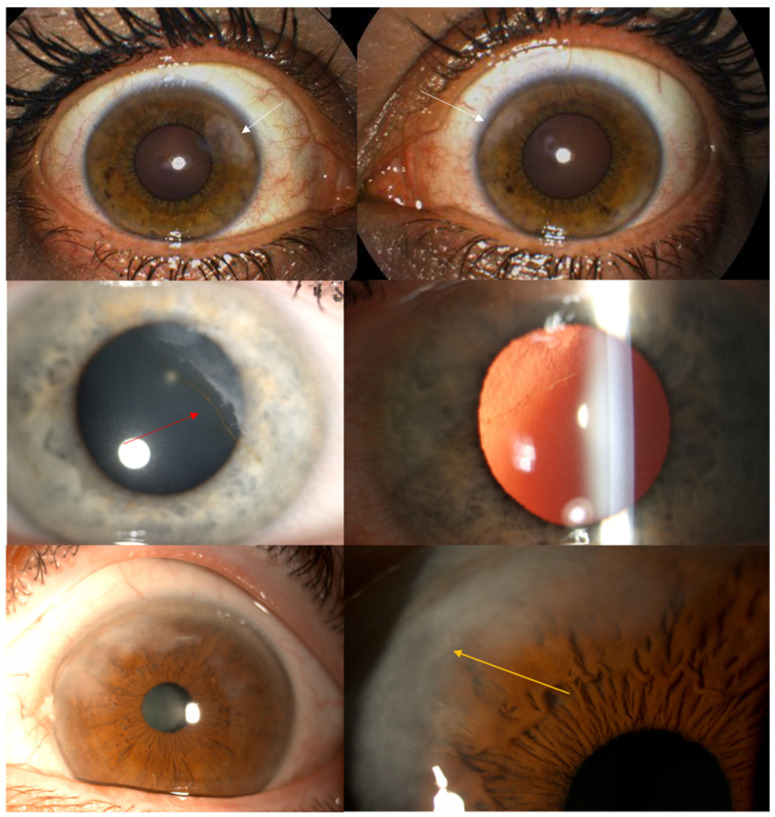
Corneal photos (first row) revealed the presence of bilateral white-greyish fibrosis located superonasally (white arrows). An iron deposit line is visible in some patients (red arrow), and is also visible under retroillumination (middle row, right image). The close-up photos (third row) show heterogeneous uneven material accumulation in the superonasal quadrant (yellow arrow). The clinical morphology of these cases is consistent with the pattern currently described as PHSCD. However, we acknowledge that an alternative classification as SND variants would be histologically defensible, given the overlapping subepithelial fibrotic features of both conditions.

**Figure 2:**
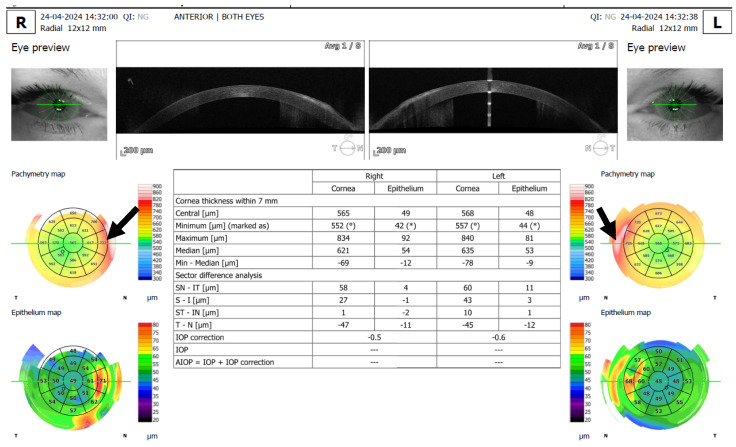
12 mm radial scan with pachymetry analysis of a patient with PHSCD showing a distinct symmetrical increase in thickness in the nasal quadrants (black arrows). The significant focal thickening in the nasal quadrants distinguishes this from smaller SND nodules, though both conditions share the underlying feature of subepithelial fibrosis. On a maps ^o^ denotes minimal thickness corneal while * denotes minimal epithelial thickness.

**Figure 3:**
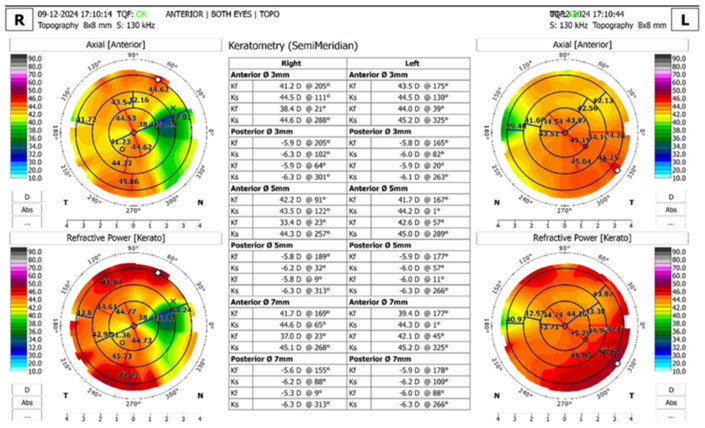
Corneal Topography Maps and Difference Map of a 64-year-old Male with a History of Peripheral Sub-epithelial Fibrosis. The topographic and tomographic findings demonstrate focal flattening at the site of peripheral fibrosis with compensatory steepening, producing irregular astigmatism. This pattern is consistent with PHSCD morphology, though SND can also produce similar topographic changes. x-denotes apex. @ stands for at median.

**Figure 4:**
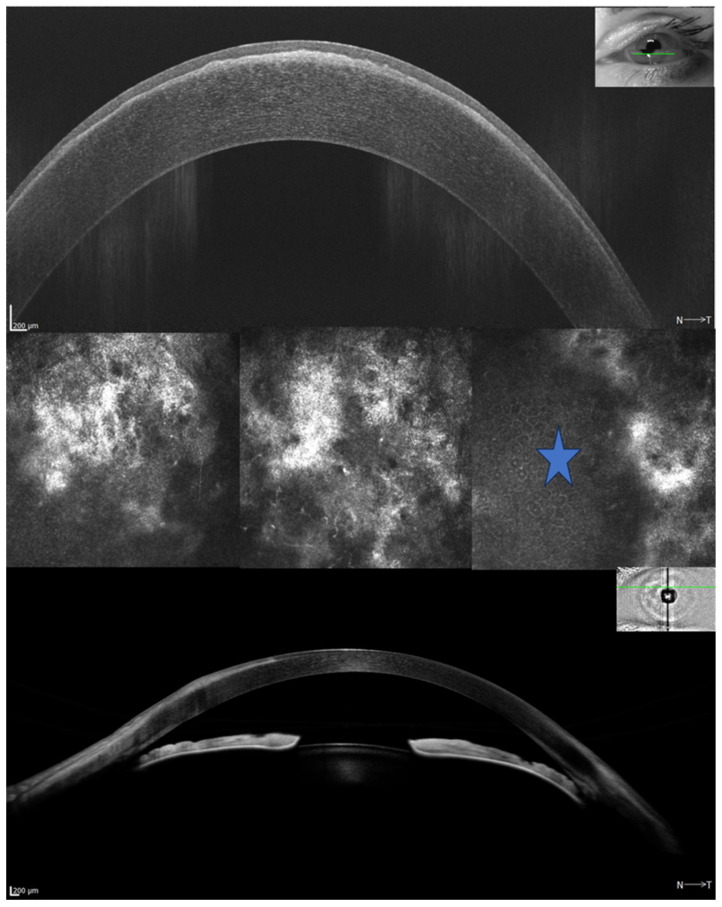
High-resolution anterior segment OCT (AS-OCT) showed peripheral hypertrophic subepithelial corneal degeneration presented with sharply demarcated uneven, hyperreflective, flat fibrosis localized within the anterior stroma. In vivo confocal microscopy revealed fibrotic acellular tissue adjacent to the normal corneal epithelium (blue star). A green line on a miniature IR image of the eye indicates the scan location.

All patients underwent an anterior segment photo (Figure 1), 12 mm × 12 mm radial scan using automatic capture mode (Figure 2), several high-resolution anterior segment line scans focused on the corneal epithelium (autofocus: 0D), using high-resolution spectral-domain optical coherence tomography with an Optopol Revo HR (Optopol Technology, Zawiercie, Poland). Corneal topography and tomography were performed using HR OCT Revo with the Topo module (Figure 3). Only scans with a QI ≥ 7 were included, and the mean QI was 7.93. In addition, three patients received in vivo confocal microscopy (HRT3, Heidelberg Engineering, Heidelberg, Germany) (Figure 4). Refraction was measured with an autorefractor (KR-1W, Topcon, Tokyo, Japan).

Cases were classified as PHSCD based on clinical morphology and imaging characteristics consistent with the pattern described in the recent literature [3,9,10]. While these cases fit the current PHSCD criteria, we recognize that classifying them as SND variants is also histologically valid due to shared subepithelial fibrotic characteristics [3,9,10]. Statistical analyses, including Spearman’s rank correlation, were performed using Statistica 13 (Tibco, Palo Alto, CA, USA).

## 3. Results

A total of 28 eyes were included in the study. The spherical error had a mean of −0.13 ± 2.49 (median −0.25, range: −5.25 to 4.00). The cylindrical error was −2.39 ± 1.48 (median −2.00, range: −6.00 to −0.50).

Various ocular parameters were assessed, including central corneal thickness, epithelial thickness, and stromal thickness, as well as sectoral differences across the corneal surface. The mean central corneal thickness was 549.4 µm (SD = 71.0 µm). Differences in sectoral thickness were analyzed in various regions, including superior-nasal (SN) to inferior-temporal (IT), superior (S) to inferior (I), superior-temporal (ST) to inferior-nasal (IN), and temporal (T) to nasal (N) regions. The mean difference in the SN-IT sector was 56.4 µm (SD = 119 µm). The ST-IN sector showed a mean difference of −16.4 µm (SD = 50.6 µm), while the T-N sector exhibited a mean difference of −49.0 µm (SD = 65.3 µm).

The mean central epithelial thickness was 54.7 µm (SD = 7.1 µm). Thickness varied across sectors, with the superior-nasal (SN_2_5) region showing a mean thickness of 57.6 µm (SD = 7.3 µm), and the temporal (T_2_5) region a mean thickness of 52.5 µm (SD = 55.5 µm).

The mean central stromal thickness was 493.5 µm (SD = 69.3 µm). Sectoral analysis revealed variations, with the SN_5_7 region having a mean thickness of 626 µm (SD = 104 µm), and the inferior-temporal (IT_5_7) region showing a mean thickness of 529 µm (SD = 80.7 µm).

Results are summarized in Table 1.

## 4. Discussion

Peripheral hypertrophic subepithelial corneal degeneration (PHSCD) is a rare, progressive condition that poses significant challenges in both diagnosis and management because of its impact on vision and its overlap with other corneal diseases.

The hallmark clinical presentation of PHSCD is a greyish, continuous subepithelial opacity that encircles the corneal periphery, typically more pronounced in the superior nasal quadrant. This peripheral fibrosis may deform the corneal surface, contributing to significant astigmatism and visual impairment [3]. Although there is no overt evidence of inflammation, some researchers hypothesize that chronic low-grade limbal inflammation may be a driving factor in the development of PHSCD by disrupting the corneal limbal barrier. Such disruption could lead to fibrotic changes in the corneal stroma, which in turn promote astigmatism.

We present the first detailed sectoral pachymetry analysis in PHSCD using high-resolution OCT with automated segmentation. Previous studies relied primarily on qualitative descriptions or central corneal thickness measurements alone. Separate corneal layer analysis indicates that both epithelial remodeling and stromal fibrosis contribute to the overall thickness profile. The comparative quadrant analysis (Figure 2) provides numerical evidence of these relationships, enhancing clinical understanding.

HR OCT showed hyperreflective, sharply demarcated subepithelial opacities. These images establish the precise anatomical location and nature of PHSCD pathology, distinguishing it from other corneal degenerations.

Our findings regarding the demographic profile of PHSCD patients align with the existing literature, confirming that this condition predominantly affects middle-aged individuals, with a higher prevalence in women. The mean age of 52.6 ± 12.4 years in our population closely mirrors previous reports that identified individuals aged 50–60 years as the primary demographic group at risk. The female predominance in our study (11 females, 3 males) supports the established gender disparity documented in previous publications.

The bilateral occurrence observed in our patients is consistent with the literature reports indicating that PHSCD typically presents as a bilateral and fairly symmetric condition. This bilaterality distinguishes PHSCD from some other corneal disorders and represents an important diagnostic criterion [1].

Quadrant pachymetry refers to the measurement of corneal thickness in the different sectors or quadrants of the cornea. In healthy corneas, thickness typically varies non-uniformly, with the cornea being thinnest centrally and progressively thicker toward the periphery.

The pathogenesis of PHSCD remains poorly understood, but emerging evidence suggests a genetic predisposition involving abnormal immune responses. Studies have implicated specific genetic markers, such as the HLA-B44 allele and the HLA-A02, B44, DRB112 haplotype, which are thought to increase susceptibility to immune-mediated fibrosis [11]. It is hypothesized that chronic, low-grade limbal inflammation may disrupt the corneal limbal barrier, leading to stromal fibrotic changes. This disruption could result in peripheral corneal deformation and subsequent irregular astigmatism. However, the absence of overt inflammation in clinical evaluations suggests that the immune mechanisms may be subtle or localized, necessitating further research to clarify these processes [4].

The observed association between PHSCD and autoimmune thyroid diseases (ATDs) in our cohort warrants careful consideration. While this represents the first systematic documentation of this relationship in PHSCD patients, similar connections have been established between ATD and other corneal surface disorders. Recent large-scale population studies have demonstrated that patients with ATDs have significantly increased risks of dry eye disease and corneal surface damage, including recurrent corneal erosions and corneal scars [12]. The pathophysiological mechanisms linking ATDs to corneal disease are multifactorial. First the systemic immune dysregulation may extend to other tissues, including the ocular surface [13]. Second, ATD is associated with lacrimal gland destruction, tear film instability, meibomian gland dysfunction, and decreased basal tear secretion [14,15]. These factors contribute to chronic ocular surface inflammation, which could trigger or exacerbate the subepithelial fibrosis characteristic of PHSCD.

However, several important limitations must be acknowledged. Our study’s small sample size and single-center design limit the generalizability of this finding. Additionally, we could not establish causality or temporal relationships—it remains unclear whether ATD precedes, occurs concurrently with, or follows PHSCD development. The high prevalence of thyroid disorders in middle-aged women (our primary demographic) may also contribute to this observation. The distinction between PHSCD and Salzmann nodular degeneration (SND) remains a subject of ongoing taxonomic debate in the corneal pathology literature. Histologically, both conditions demonstrate similar features: subepithelial fibrosis, disrupted or absent Bowman’s layer, thin overlying epithelium, and activated fibroblasts. However, morphological and clinical patterns suggest possible clustering that may distinguish PHSCD from classical SND presentations (Figure 4) [1]. Currently described distinguishing features of PHSCD include: (1) larger, more continuous arcuate or circumferential subepithelial fibrosis (typically >5–10 mm) located in perilimbal regions, particularly the superior-nasal quadrant, compared with SND typically smaller (1–3 mm), discrete nodules distributed more randomly in the mid-periphery; (2) greater bilateral symmetry; (3) associated superficial corneal neovascularization extending from the limbus [16]; (4) less frequent association with preceding surgical trauma or chronic irritation; and (5) possible association with systemic autoimmune conditions.

These nodules are associated with a variety of systemic and ocular risk factors, including dry eye disease, ocular surface inflammation, and androgen deficiency, which may explain the higher prevalence of SND in women [17]. Additionally, SND has been linked to surgical interventions such as LASIK, cataract extraction, and corneal transplants, where post-surgical dry eye or trauma may act as triggering mechanisms [18]. Systemic conditions like Crohn’s disease have been implicated as potential triggers. In contrast, PHSCD presents as a continuous fibrotic opacity rather than discrete nodules and does not exhibit a clear association with surgical or traumatic events. Importantly, we acknowledge that these morphological differences represent patterns of presentation rather than evidence of fundamentally distinct underlying pathophysiology. The histological overlap is substantial, and experienced clinicians could reasonably classify some cases of PHSCD as SND variants. Further molecular, genetic, and biomechanical studies are needed to clarify whether these represent distinct disease entities or a spectrum of subepithelial fibrotic responses with different triggering mechanisms and clinical presentations

No single imaging finding is pathognomonic for PHSCD vs. SND. The distinction rests on pattern recognition rather than a definitive structural difference. AS-OCT imaging revealed the morphological pattern of PHSCD as a continuous, well-demarcated, hyperreflective subepithelial band located peripherally and with a superior-nasal predominance, a pattern that differs from the more randomly distributed, smaller nodules (1–3 mm) typically described in Salzmann nodular degeneration [10]. Topographic and tomographic evaluations demonstrated focal corneal flattening at sites of fibrosis with compensatory steepening in opposing quadrants, producing characteristic irregular astigmatism patterns.

In PHSCD, the deposition of subepithelial fibrotic tissue increases corneal thickness in affected regions, predominantly in the superior-nasal quadrant. This focal thickening alters the corneal curvature through two mechanisms. First, the added mass of fibrotic tissue mechanically flattens the underlying cornea, reducing its curvature and refractive power in that meridian [19]. Second, the non-uniform thickness distribution creates an imbalance in corneal biomechanical forces, leading to compensatory steepening in opposing meridians to maintain overall corneal integrity [20].

Contrary to our initial hypothesis, we did not observe a statistically significant linear correlation between the SN-IT thickness gradient and the magnitude of cylindrical error. This finding likely reflects the complex, non-linear biomechanical interactions in PHSCD pathophysiology. The cornea responds to focal thickening through compensatory mechanisms that may not follow simple linear relationships. Interestingly, the ST-IN thickness gradient showed a significant correlation with astigmatism, suggesting that meridian orientation relative to the fibrosis may influence the structure–function relationship.

The increased epithelial thickness overlying fibrotic regions likely represents a compensatory remodeling response. The epithelium exhibits remarkable plasticity and changes thickness to smooth underlying irregularities—a phenomenon well-documented in other corneal conditions such as SND, keratoconus, and post-refractive surgery ectasia [21].

In PHSCD, this compensation is incomplete, as the epithelial thickness increases we measured (approximately 5 μm) were insufficient to counterbalance the much larger stromal thickness increases (mean 56 μm in SN-IT gradient), resulting in persistent irregular astigmatism.

Although the present study focused on diagnostic characterization rather than treatment outcomes, it is important to acknowledge the management context. Seven patients (50%) in our cohort required PTK intervention due to progressive visual impairment from irregular astigmatism. The exclusion of treatment outcomes from this report was intentional to maintain focus on establishing comprehensive baseline imaging characteristics and on varied follow-up periods.

## 5. Conclusions

The diagnosis of PHSCD is primarily clinical, based on characteristic clinical signs, including irregular astigmatism, topographic flattening, and subepithelial fibrosis with peripheral vascularization [4]. These findings are used in the diagnosis of PHSCD and include: (1) grey-colored continuous subepithelial fibrosis leading to regular and irregular astigmatism; (2) corneal flattening based on topographical mapping; and (3) typical revascularization in the peripheral region of the fibrous tissue.

Second-level examinations helpful for PHSCD diagnosis include topography, tomography, anterior segment OCT (AS-OCT), and confocal microscopy. Topographic and tomographic evaluations play an essential role in identifying corneal irregularities not only in PHSCD but also across a wide spectrum of corneal pathologies [22].

PHSCD is a rare, progressive corneal condition that predominantly affects middle-aged women. The characteristic peripheral subepithelial fibrosis causes significant variations in corneal thickness and astigmatism, affecting visual acuity. The association with thyroid disorders, particularly Hashimoto’s disease, suggests an immunological component in the pathogenesis of PHSCD, warranting further investigation. PHSCD poses a significant risk of progressive visual impairment due to its impact on the corneal structure. The resulting corneal flattening in affected meridians often induces hyperopic shifts and irregular astigmatism. Early recognition is critical to prevent further visual decline.

## Figures and Tables

**Table 1:** Summary of the pachymetry findings.

Variable	Mean	Median	Minimum	Maximum	Std. Dev.
Central [µm]	549.4	548.5	440.0	768.0	71.0
Minimum [µm]	521.4	521.0	428.0	631.0	48.8
Median [µm]	595.5	608.1	489.0	701.0	51.9
Min-Median [µm]	−74.2	−64.0	−149.0	−40.0	27.7
SN-IT [µm]	56.4	51.0	−277.0	333.0	119.0
S-I [µm]	19.9	33.0	−253.0	175.0	93.6
ST-IN [µm]	−16.4	−7.0	−145.0	78.0	50.6
T-N [µm]	−49.0	−44.5	−152.0	139.0	65.3
Epithelium thickness					
Central [µm]	54.7	54.0	42.3	68.0	7.1
Minimum [µm]	41.8	42.5	26.0	57.8	7.2
Median [µm]	50.3	56.0	−14.2	61.0	18.6
Min-Median [µm]	−12.8	−13.4	−29.0	13.0	9.0
Sectors difference analysis					
SN-IT [µm]	2.5	1.0	−7.0	15.0	5.7
S-I [µm]	−0.6	−2.0	−24.0	16.0	8.4
ST-IN [µm]	−3.0	−3.0	−14.0	13.0	5.7
T-N [µm]	−6.8	−6.0	−25.0	6.0	7.9

Correlation analysis between the SN-IT thickness gradient and the magnitude of cylindrical error revealed no statistically significant relationship (Spearman ρ = 0.274, *p* = 0.418). However, analysis of the S-I (Spearman ρ = 0.467, *p* = 0.033) ST-IN thickness gradients revealed a significant moderate positive correlation with absolute cylindrical error (Spearman ρ =0.514, *p* = 0.017).

## Data Availability

The original contributions presented in this study are included in the article. Further inquiries can be directed to the corresponding author.

## Abbreviations

The following abbreviations are used in this manuscript:

PHSCD	Peripheral hypertrophic subepithelial corneal degeneration
OCT	Optical coherence tomography
AS-OCT	Anterior segment optical coherence tomography
HR OCT	High-resolution optical coherence tomography
SD	Standard deviation
SND	Salzmann’s nodular degeneration
ACSH	Acquired corneal sub-epithelial hypertrophy
PTK	Phototherapeutic keratectomy
FP	Fibrous peeling
BCVA	Best-corrected visual acuity
PRK	Post-photorefractive keratectomy
CCT	Central corneal thickness
ATD	Autoimmune thyroid diseases
LASIK	Laser-Assisted in situ Keratomileusis
